# Drop tower tests of Taiji-1 inertial sensor substitute

**DOI:** 10.1038/s41526-021-00154-8

**Published:** 2021-07-07

**Authors:** Jian Min, Zuo-Lei Wang, Yun-Peng Li, Wen-Ze Tao, Cun-Hui Li, Jun-Gang Lei, Dong-Xue Xi, Da Fan, Jun-Biao Wang

**Affiliations:** 1 National Key Laboratory of Science and Technology on Vacuum Technology and Physics, Lanzhou Institute of Space Technology and Physics, Lanzhou, China; 2 Qian Xuesen Laboratory of Space Technology, Beijing, China; 3 National Microgravity Laboratory, Institute of Mechanics, Chinese Academy of Sciences, Beijing, China

**Keywords:** Characterization and analytical techniques, Electrical and electronic engineering, Aerospace engineering

## Abstract

Taiji-1, which is the first technical verification satellite of China’s Space Gravitational Wave Detection Program, was successfully launched on August 31, 2019. The mission aimed to investigate the key technologies used in space gravitational wave detection. The inertial sensor, which was one of the main payloads, measured the residual acceleration of the satellite, and verified the drag-free control technology. Its performance was crucial to the success of the Taiji-1 mission. To ensure its performance in orbit, the inertial sensor was fully evaluated prior to launch. Owing to the gravitational acceleration on the ground, it is impossible to verify all the properties of the inertial sensor in a routine laboratory. A feasible method to conduct such tests is to use a drop tower. To guarantee the safety of the inertial sensor, a substitute was used with similar structure and circuit design. A total of 20 falls in three groups were completed, a set of research methods was established, and the importance of conducting simulations before the drop tests was verified. For the first time, the switch of different circuit gains in a drop tower test has been achieved and the National Microgravity Laboratory of China (NMLC) drop tower’s residual accelerations in three dimensions were measured. The results demonstrated that the microgravity level of the drop tower can reach about 58 μg_0_ in the fall direction and 13 μg_0_ along the horizontal axes.

## Introduction

Following the success of ground-based gravitational wave (GW) detection^1,2^, space-based GW detection offers a novel method for studying the universe^3–6^. Compared to LIGO-like GW detectors on the ground, LISA-like GW detectors in space aim for a lower frequency range of 0.1 mHz~1 Hz. The Taiji program is one of China’s space GW exploration programs and is planned to launch around 2033^7,8^. An inertial sensor will be used as a position reference for both drag-free control and laser interference technologies. To ensure the success of the program, its accuracy needs to be 5~10 pm/$\sqrt {Hz}$^9^.

**Fig. 1:**
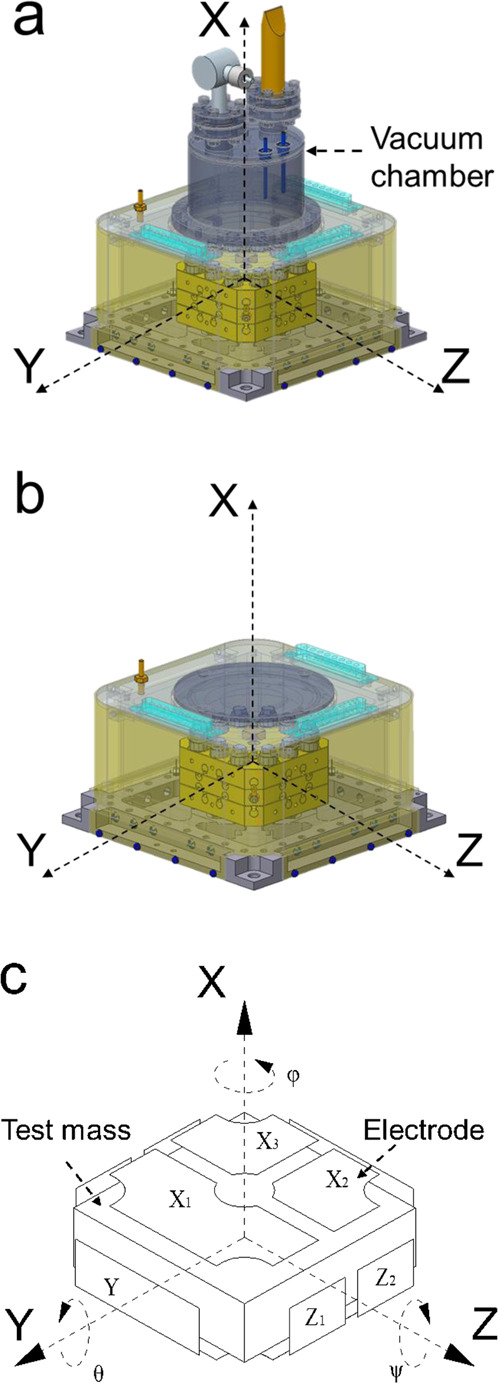
The SIS and the inertial sensor. **a** Schematic diagram of the SIS. **b** Schematic diagram of the actual inertial sensor. **c** Axial diagram of the electrode housing.

The first technical verification satellite of the Taiji program, known as Taiji-1, was launched on August 31, 2019^10^. One of the main payloads on Taiji-1^11–13^ is the inertial sensor. To estimate its performance, a series of ground tests needs to be conducted. However, due to the gravitational acceleration in the direction of gravity, it is almost impossible to fully evaluate the in-orbit performance of the inertial sensor in a routine laboratory. Furthermore, in order to improve the stability of the control system and achieve higher measurement accuracy, the circuit gains need to be switched at times. However, this cannot be performed on the ground with a high-voltage suspension along the X-axis (Fig. 1) (i.e., the direction of gravity). For the above reasons, it is important to simulate the in-orbit performance with the inertial sensor prior to launch. An appropriate solution is to use a drop tower to simulate a microgravity environment and then conduct systematic tests.

For both the Aster and GOCE accelerometers, the ZARM drop tower was used for acceptance testing^14,15^. For the MICROSCOPE accelerometer, the ZARM drop tower was also used to demonstrate the control capability of the two coaxial test masses^16,17^. In December 2016, researchers conducted a series of drop tower tests with a reduced prototype of MicroSTAR at ZARM to verify the concept and optimize the accelerometer control loop^18^.

For the experiments presented in this paper, the drop tower of the National Microgravity Laboratory of China (NMLC) in Beijing was used, which is capable of providing a free-fall time of about 3.5 s and a microgravity level of about 10^−5^ g_0_, where g_0_ is the local gravitational acceleration^19^. Two groups of mode-switch experiments were performed and three different control modes were used. These experiments allowed not only the performance evaluation of the inertial sensor, but also demonstrated the effect of circuit gain switching on the control loop under microgravity environment.

To ensure the safety of the real inertial sensor, a substitute inertial sensor (SIS) was used in the drop tower test. The SIS had almost the same structure with the actual inertial sensor, the only difference being the vacuum device on the top of the sensor-head. The SIS had a vacuum chamber to maintain a vacuum on the ground (Fig. 1a), while in the in-orbit sensor, this chamber was removed and the vacuum in space was used (Fig. 1b). Moreover, the SIS had the same circuit design as that of the inertial sensor on Taiji-1, and in order to adapt to the microgravity level of the drop tower, the gain of the amplifier circuit was adjusted. The electrode housing and the axial diagram of the sensor-head are illustrated in Fig. 1c. There were three electrode pairs along the X-axis, two along the Z-axis, and one along the Y-axis.

A cuboid-shaped gold-plated titanium alloy was used as the test mass to perceive the acceleration of the sensor. The test mass was connected to the measure and control unit (MCU) through a gold wire to provide it with a DC bias voltage (*V*_p_) and a position detection AC signal (*V*_d_). The electrode housing surrounding the test mass was made of gold-plated microcrystalline glass, taking into consideration the thermal stability and magnetic susceptibility of the sensor. With the vacuum sealing structure, the vacuum pressure of the SIS could be maintained below 10^−5^ Pa on the ground.

The displacement signal of the test mass was obtained by measuring the capacitance difference between test mass and electrodes. At the same time, a PID controller was used to ensure that the test mass was positioned at the center of the electrode housing, and the acceleration of the sensor-head was recorded through the feedback voltages. The average value of the displacement or acceleration along each axis was used to describe the translation of the sensor-head, and the corresponding difference value was used to represent the rotation of the sensor-head.

## Results

### Brief summary

In total, 20 drop tests were conducted and a complete set of test methods was established, including a simulation model and a way to control the simulation precision. For the first time, a switch of different circuit gains was achieved in a drop tower test. As long as the control parameters were set to reasonable values, the effect of circuit gain switching on system stability was negligible. These results provide an important experimental reference for the in-orbit operation of the inertial sensor on the Taiji-1 satellite. Furthermore, the NMLC drop tower’s residual accelerations in three dimensions were measured. In the last 0.5 s of the free-fall, the lowest residual acceleration reached about 58 μg_0_ along the X-axis (the falling direction) and 13 μg_0_ along the Y- and Z-axes. The experimental results in this paper also have important reference value for researchers who plan to conduct experiments using the NMLC drop tower.

### Control modes

The different control modes were named as capture mode, large-range mode, and small-range mode. The voltage parameter settings for the different control modes are given in Table 1.

**Table 1:** Voltage settings in the different control modes.

Control mode	Position detection voltage (V)	Bias voltage (V)	Maximum feedback voltage (V)
Capture mode	1	55	35
Large-range mode	4	55	35
Small-range mode	4	30	17.5

Due to the fact that the duration of the free-fall was only about 3.5 s, the time to switch from the capture mode to the large-range mode, and then to the small-range mode at one single drop was not sufficient. To this end, two sets of experiments were designed; a large-range mode switching experiment and a small-range mode switching experiment. Each experiment involved a change from capture mode to large- or small-range mode, respectively.

### Modes switching

**Fig. 2:**
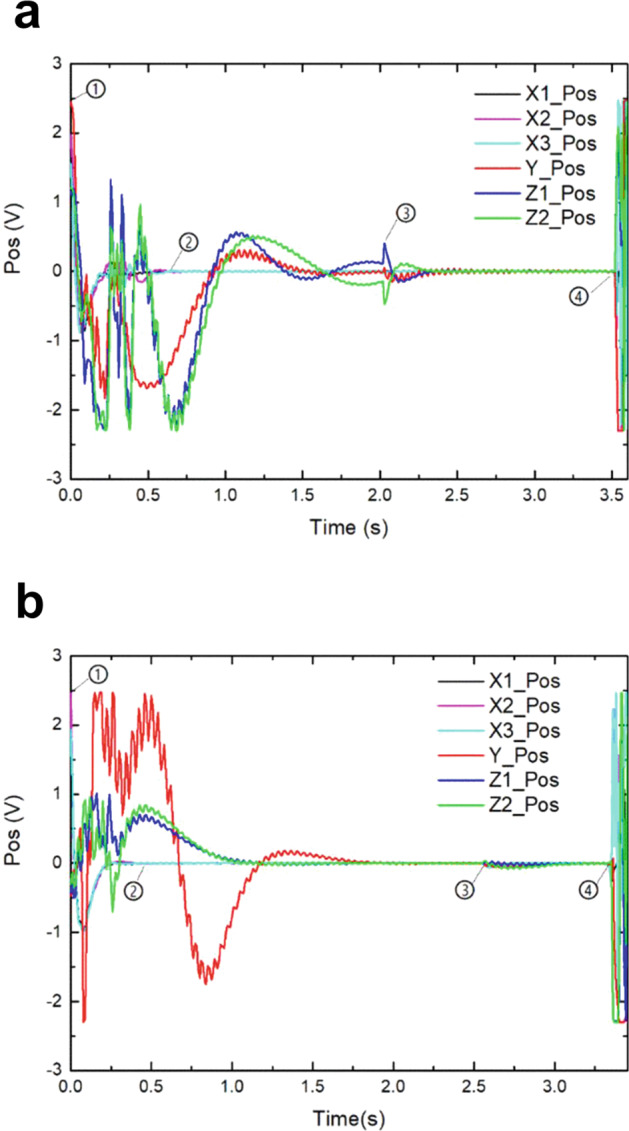
Displacement voltages of all axes. In both large- and small-range modes (**a**, **b**), the outer capsule began to fall at ①, the test mass along the X-axis reached the center of the electrode housing at ②, and the mode switch took place at ③. The free-fall ended at ④ due to collision between the inner and outer capsules.

The displacement voltage curves for each direction during the free-fall in the large-range mode switching experiment are shown in Fig. 2a. Before the free-fall, the SIS rested on mechanical stops, and the displacement voltages along each axis remained constant. At 0 s, the outer capsule began to fall, and after 0.64 s (from ① to ②), the test mass of the SIS along the X-axis was controlled to the center of the electrode housing. The displacement curves in the horizontal axes (Y- and Z-axes) converged more slowly, requiring about 1.5 s to converge to zero. The mode switch took place at ③ and caused a voltage overshoot of up to 0.3 V along the Z1 and Z2 axes. Then, the SIS switched to the large-range mode and the test mass reached the center of the electrode housing within 0.5 s. The microgravity environment of the inner capsule disappeared at ④ (3.52 s) and the displacement voltages of each axis changed dramatically due to the collision between the inner and outer capsules.

The displacement voltage curves for each direction during the free-fall in the small-range mode switching experiment are shown in Fig. 2b. The displacement voltages along the X-axis converged within 0.44 s (from ① to ②), while it took a longer time for the Y- and Z-axes to converge to zero. At point ③, the SIS switched to small-range mode, causing a vibration of only about 0.1 V. Then, the test mass reached the center of the electrode housing within 0.2 s along the X-axis and within about 0.75 s along the horizontal axes. The free-fall lasted about 3.32 s (from ① to ④).

## Discussion

**Fig. 3:**
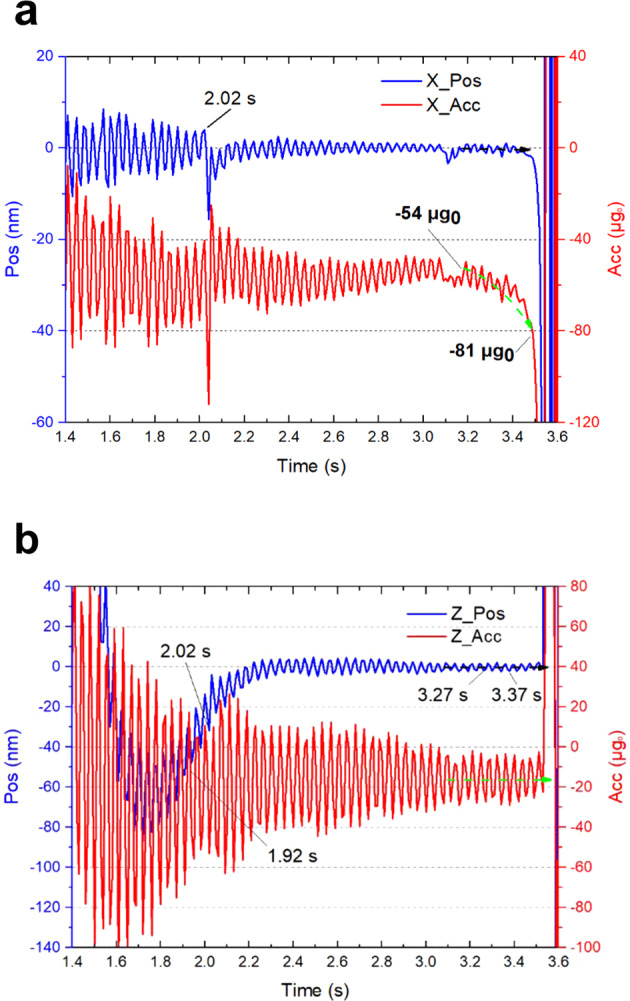
Magnified view of the measured curves in the large-range mode switching experiment. The displacement (blue) and acceleration (red) along the X- and Z-axes are shown in (**a**) and (**b**), respectively. The green dashed arrows indicate the residual acceleration while the black dashed arrows indicate the displacement at the end of the free-fall.

The translational motion of the SIS was determined by calculating the average displacement and acceleration along each axis. The displacement and acceleration along the X-axis are presented in Fig. 3a. The mode switching took place at 2.02 s and the displacement-detection gain was reduced by four times compared to that in capture mode. The PID parameters changed accordingly. This induced a displacement vibration of about 20 nm only and an acceleration overshoot of about 87 μg_0_, which was attributed to the change of the electrostatic forces applied to the test mass. At the end of the free-fall, a significant increase in acceleration occurred (green dashed arrow), while the test mass was maintained at the center of the electrode housing (black dashed arrow) until the last 0.1 s.

A magnified view of the displacement and acceleration along the Z-axis before and after mode switching is presented in Fig. 3b. As indicated by the dashed arrows, both the displacement and acceleration along the Z-axis remained stable until the last 0.1 s. This is in sharp contrast to the X-axis data presented in Fig. 3a. This difference may have been induced by the air resistance during the free-fall or by the interaction between the inner and outer capsules during the end of the free-fall.

The air-drag force can be expressed as Eq. (1):^20^

1 $$F = \frac{1}{2}C_{\mathrm{D}}\rho Sv^2$$

where *F* is the air-drag force, *C*_D_ is the drag coefficient, *ρ* is the air density, *S* is the cross-sectional area of the capsule, and *v* is the velocity relative to the atmosphere.

The residual acceleration of the inner capsule can be expressed as Eq. (2):^20^

2 $$a_{\mathrm{r}} = {\mathrm{g}}_0{\mathrm{tanh}}^2\sqrt {\frac{{C_{\mathrm{D}}\rho S{\mathrm{g}}_0t}}{{2m}}}$$

**Fig. 4:**
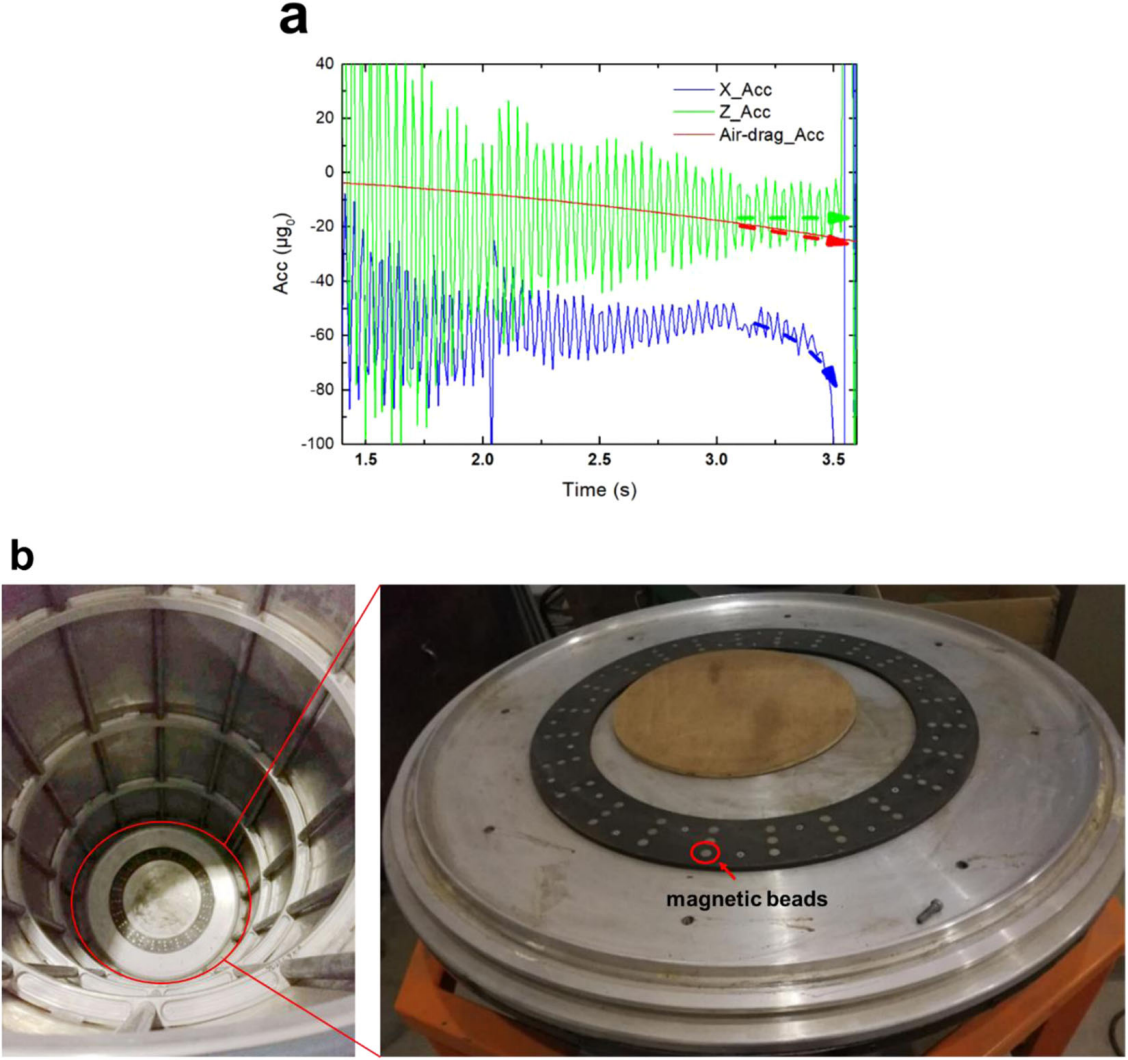
Analysis of the end of free-fall. **a** Comparison between the acceleration induced by air drag (red) and that measured along the X- and Z-axes (blue and green) in the large-range mode switching experiment. **b** Top view of the outer capsule (left) and closer view of the bottom of the outer capsule, where the distribution of the magnetic beads can be observed (right).

Based on the parameters of the inner capsule (i.e., *m* = 217 kg, *C*_D_ = 0.2, *ρ* = 1.15e-3 kg/m^3^, and *S* = 0.385 m^2^), the acceleration induced by air drag and that measured along the X- and Z-axes in the large-range mode switching experiment were compared, and the comparison results are presented in Fig. 4a. Compared with the rapid acceleration change along the X-axis in the last 0.3 s, the amplitude of the air-drag acceleration was much smaller and increased more slowly. Therefore, it can be confirmed that the rapid acceleration change along the X-axis was not induced by the air resistance.

Seventy-two magnetic beads were placed at the bottom of the outer capsule (Fig. 4b), which were used to attract and fix the inner capsule at the end of the free-fall, and prevent the collision between the outer and inner capsules after contacting the deceleration unit.

At the last 0.3 s of the free-fall, the inner capsule reached near the bottom of the outer capsule before the latter touched the deceleration unit. Consequently, it can be inferred that the 72 magnetic beads attracted the inner capsule and induced a large acceleration change along the X-axis.

The data within 0.1 s in the most stable period in both the capture mode (1.92~2.02 s) and the large-range mode (3.27~3.37 s) exhibited the minimum displacement and lowest acceleration. The average value and maximum fluctuations were calculated, and are shown in Table 2.

**Table 2:** Comparison of displacement and acceleration data in the large-range mode switching experiment.

Control mode	Test item	Displacement (nm)		Acceleration (μg_0_)	
		X	Z	X	Z
Capture mode	Average value	0.3	−30.4	−59.3	−20.5
	Maximum fluctuations	8.6	34.3	38.2	73.3
Large-range mode	Average value	−0.3	−0.1	−59.4	−15.5
	Maximum fluctuations	3.1	4.5	12.8	25.6
Largest vibration during mode switching		20	28	87	63

After mode switching, the average acceleration along the Z-axis was reduced from −20.5 to −15.5 μg_0_ and the acceleration fluctuation was reduced by about three times from 73.3 to 25.6 μg_0_, indicating that the test mass was further controlled and the average displacement reached −0.1 nm only (Table 2).

**Fig. 5:**
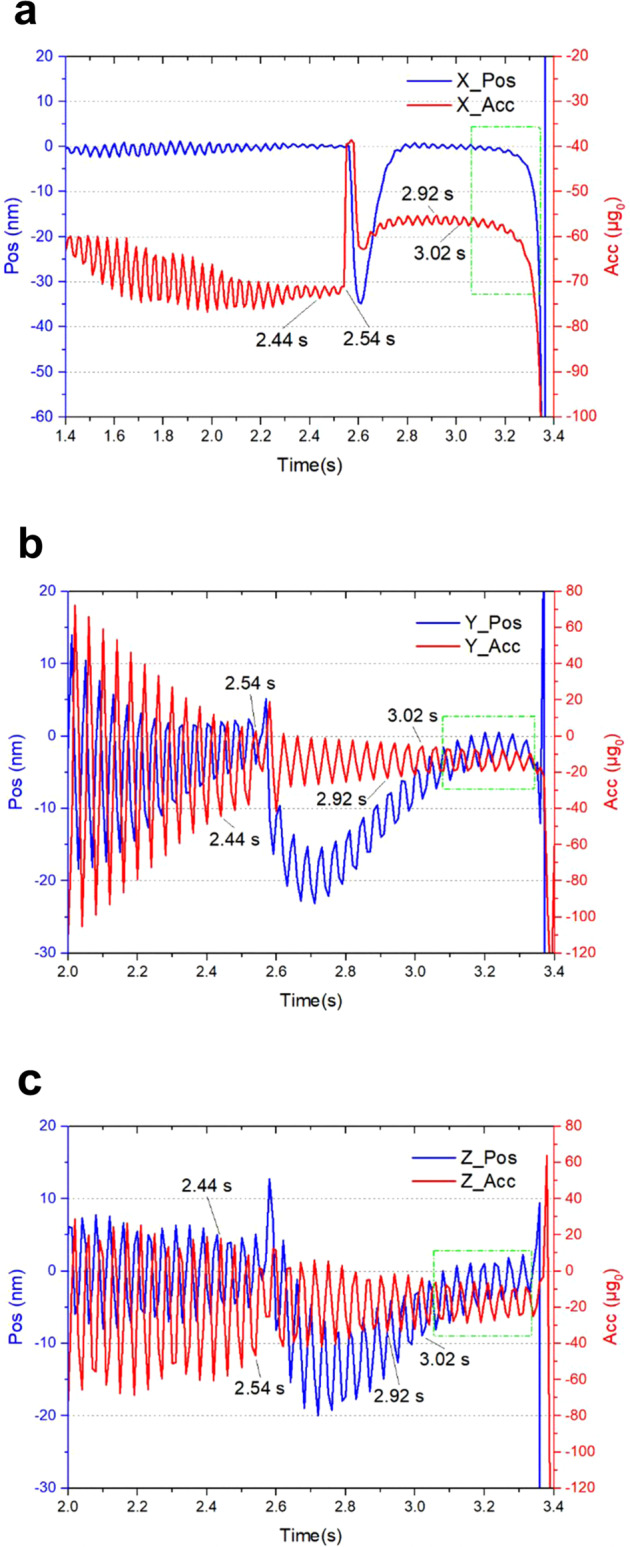
Magnified view of the measured curves in the small-range mode switching experiment. The displacement (blue) and acceleration (red) along X-, Y-, and Z-axes are shown in (**a**), (**b**), and (**c**), respectively. The green dashed box indicates the end of the free-fall.

In the small-range mode switching experiment, the mode switch of the X-axis took place at 2.54 s, producing an obvious vibration (Fig. 5a). The displacement-detection gain was reduced by four times and the feedback-loop gain was reduced by half after the switch to small-range mode.

The magnified views of Y- and Z-axes are illustrated in Fig. 5b, c, respectively. The displacement and acceleration data along both axes remained stable before the end of the free-fall, as indicated by the green boxes.

The data along the three axes within 0.1 s in the most stable period in the capture mode (2.44~2.54 s) and small-range mode (2.92~3.02 s) are presented in Table 3.

**Table 3:** Comparison of displacement and acceleration data in the small-range mode switching experiment.

Control mode	Test item	Displacement (nm)			Acceleration (μg_0_)		
		X	Y	Z	X	Y	Z
Capture mode	Average value	0.1	−1.0	−0.3	−72.0	−16.4	−21.2
	Maximum fluctuations	0.7	7.5	10.1	2.7	52.5	76.3
Small-range mode	Average value	−0.01	−8.1	−7.2	−56.4	−13.1	−17.6
	Maximum fluctuations	1.0	7.7	9.8	2.0	19.5	31.7
Largest vibration during mode switching		35	28	33	32	61	48

Based on the average acceleration of the X-axis in both large- and small-range modes, we conclude that during the free-fall test, the minimum residual acceleration along the X-axis was about −58 μg_0_ and about −13 μg_0_ along the horizontal axes. Furthermore, the largest acceleration vibration was about 87 μg_0_ (Table 2) and the displacement vibration was only about 35 nm (Table 3) during mode switching, indicating that when the control parameters are set properly, the effect of circuit gain switching on system stability is negligible.

The inner capsule was equipped with a uniaxial quartz-accelerometer (QA), which can measure the acceleration along the falling direction. The resolution of the QA was 4.675 mg_0_, and the measurement range was ±7.7 g_0_. The acceleration measured by the QA can record the state of the inner capsule during the free-fall.

**Fig. 6:**
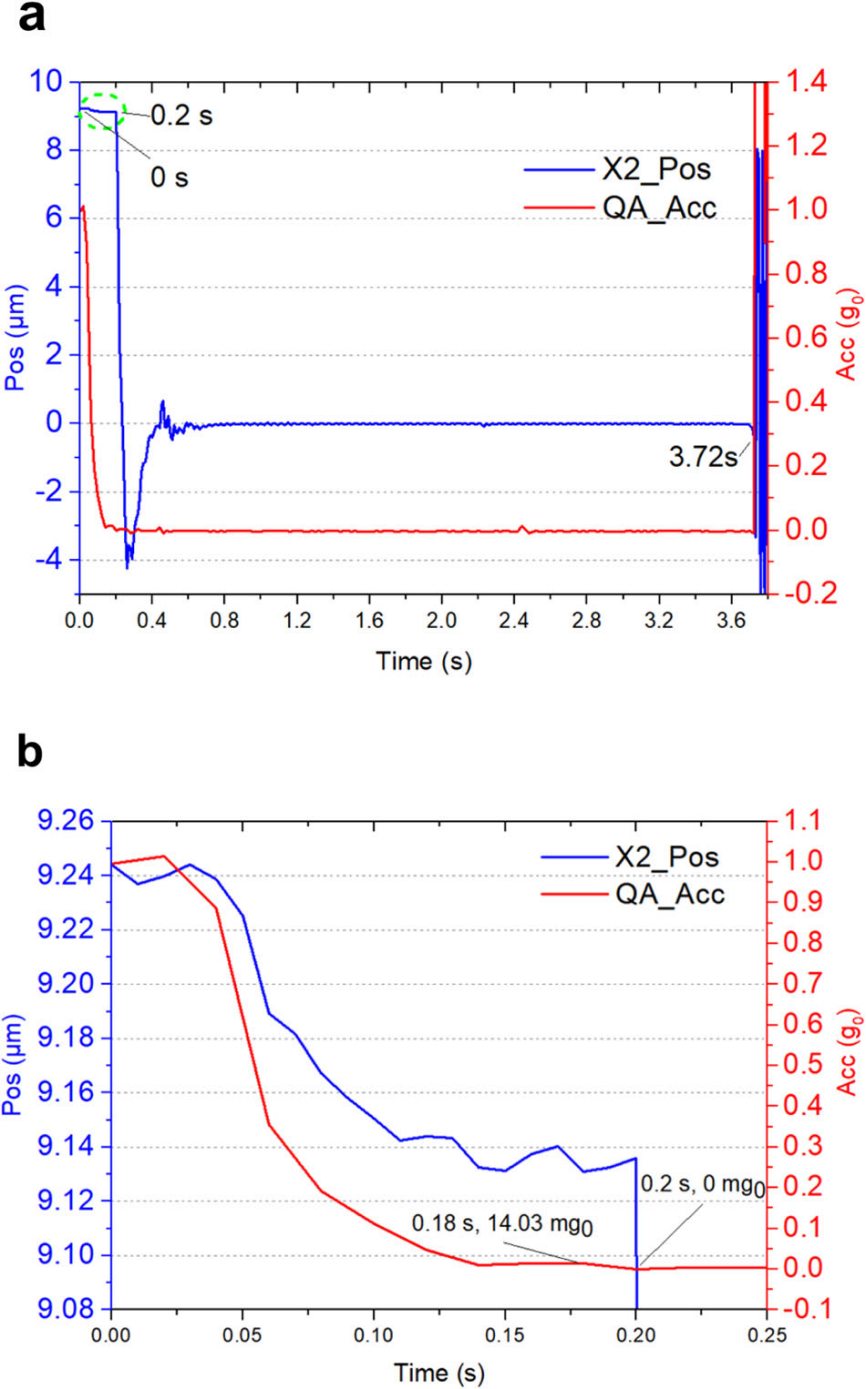
Data comparison between the displacement along the X2 axis and the acceleration of the QA in the large-range mode switching experiment. **a** Data of the X2 axis (blue) and the QA (red) during the free-fall. **b** Magnified view at the beginning of the release of the outer capsule (0~0.25 s).

The displacement data measured by the SIS along the X2 axis and the acceleration data measured by the QA in the large-range mode switching test were chosen for comparison (Fig. 6a, b).

Before 0 s, the microgravity level measured by the QA along the falling direction was about 1 g_0_, while the displacement voltage of the SIS was at a saturation state, indicating that the inner capsule was stationary at the top of the tower. As soon as the outer capsule was released at 0 s, the acceleration measured by the QA decreased rapidly towards zero (Fig. 6a). Meanwhile, the displacement of the test mass changed by only about 0.1 μm in the first 0.2 s, which was caused by the vibration generated during the release of the inner capsule. Although the residual acceleration decreased rapidly during the first 0.2 s of the free-fall, it still exceeded the highest capture-acceleration in the X-axis of the SIS (9.6 mg_0_; Table 4) and the test mass could not reach the center of the electrode housing. At 0.2 s, the acceleration measured by the QA reached zero (Fig. 6b), indicating that the residual acceleration was less than 4.675 mg_0_ (resolution of the QA), and lower than the capture-acceleration of the SIS along the X-axis; thus, the test mass moved quickly towards the center of the electrode housing.

**Table 4:** Comparison of the range and maximum operational acceleration (mg_0_).

Axis	SIS			Inertial sensor	
	Maximum operational acceleration (mg_0_)	Range		Maximum operational acceleration (mg_0_)	Range
		Large-range mode	Small-range mode		
X	9.60	15.0	4.30	1.60	0.04
Y	0.93	1.6	0.46	0.18	0.005
Z	0.93	1.6	0.46	0.18	0.006

Owing to the disturbance during the release of the capsule, the residual acceleration along the X-axis would be higher than 10 mg_0_ in a short time (e.g., 0.2 s), and the test mass could not be captured in this period. A conclusion that can be drawn is that in order to achieve the longest free-fall time in a drop tower test, a stable release process with minimum residual acceleration should be ensured. For instance, under the premise of synchronous release of the outer and inner capsules, experiments should not be performed in the case of strong external winds (greater than 10 m/s) in order to avoid additional disturbance from the drop tower. In addition, strong rigid connections should be used for the inner capsule to reduce the vibration after the release. At the same time, all equipment and cables should be firmly fastened to the capsule.

**Fig. 7:**
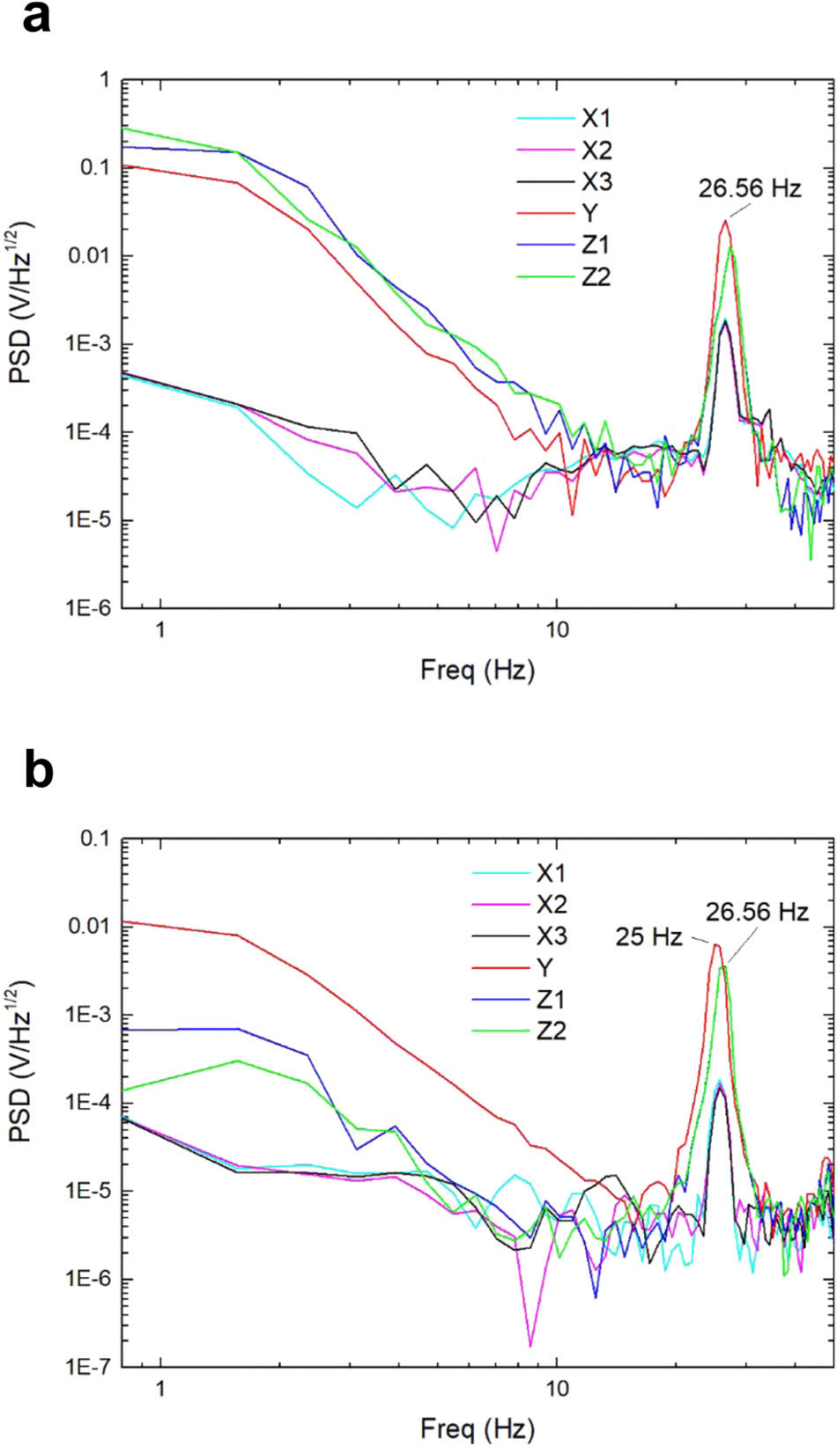
Spectrum analyses. Resonance peak of the SIS displacement voltages in the large- and small-range mode switching experiments (**a**, **b**).

Spectrum analyses for both the large- and small-range mode switching experiments were performed. The SIS displacement voltages in the capture mode were used and the results are shown in Fig. 7a, b.

The acceleration of the inner capsule increased abruptly from 0 to 1 g_0_ when the electromagnetic lock powered off, which caused the inner capsule to vibrate near its natural resonant frequency. As can be seen in Fig. 7a, b, there were apparent resonance peaks around 26.56 Hz in each axial direction, which reflect the natural resonant frequency of the inner capsule.

## Methods

### NMLC drop tower and experimental equipment

**Fig. 8:**
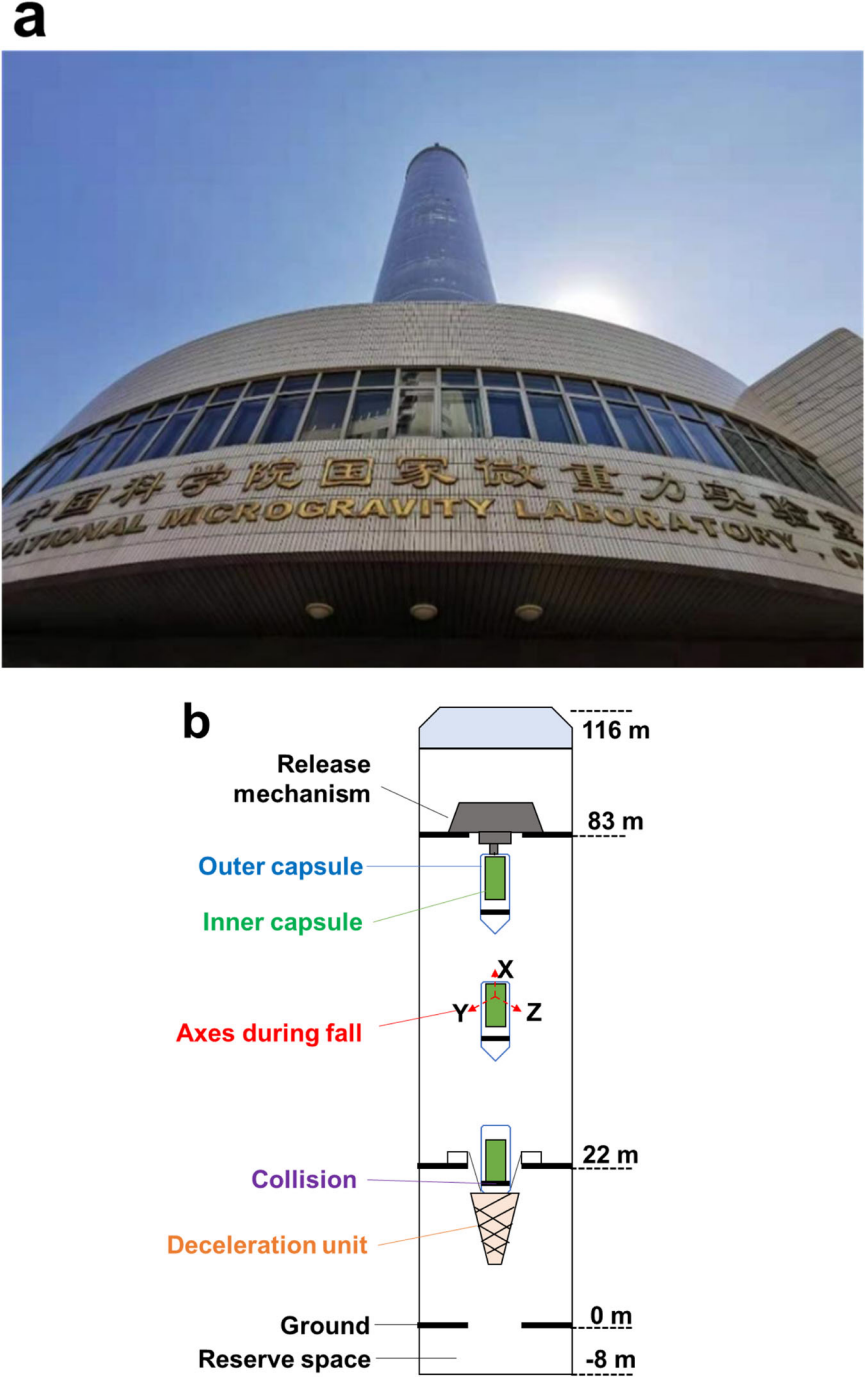
The NMLC drop tower. **a** An upward view of the building. **b** Sketch of the drop tower’s experimental configuration.

The drop tower of the NMLC in Beijing (Fig. 8a) has a total height of 124 m, including 116 m above the ground and 8 m below the ground. The total free-fall distance is about 60 m, providing a free-fall time of about 3.5 s. Figure 8b illustrates a sketch of the drop tower experimental configuration. The X-axis of the SIS was parallel to the longitudinal axis of the inner capsule, while the Y- and Z-axes of the SIS were perpendicular to the X-axis.

There are two installation modes in a drop tower test, namely, the Single Capsule Mode and the Double Capsule Mode. The microgravity level during the fall is about 10^−3^ g_0_ in the Single Capsule Mode and can reach about 10^−5^ g_0_ in the Double Capsule Mode^19,21^. For the experiments in this paper, the Double Capsule Mode was adopted.

### Drop tower test procedure

**Fig. 9:**
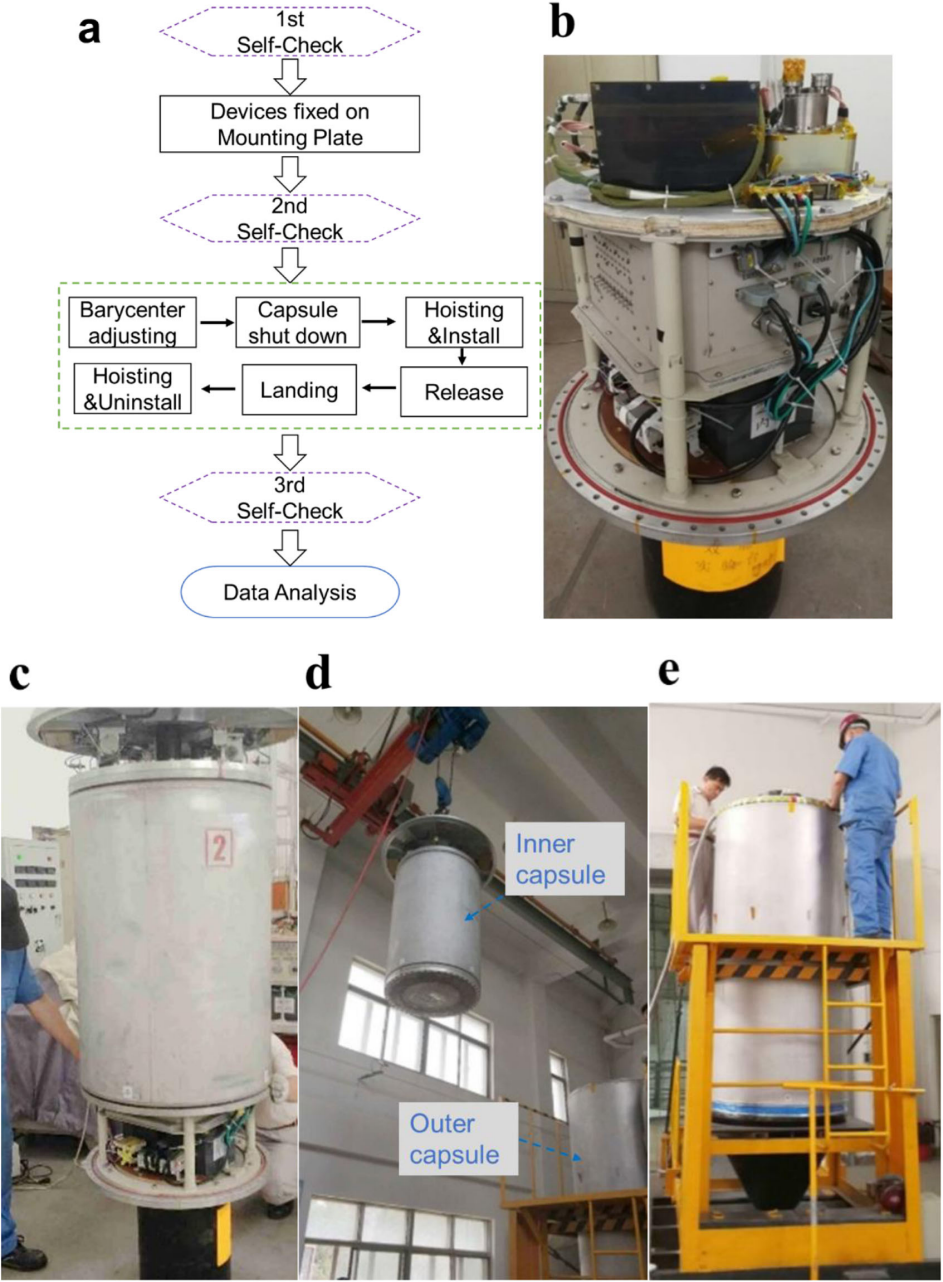
Drop tower test procedure. **a** Flowchart of the drop tower test. **b** Devices installed on the mounting plate. **c** Sealed inner capsule for barycentric correction. **d** Lifted inner capsule into the outer capsule. **e** Outer capsule pumped below 30 Pa and sealed.

The basic steps of the experimental process are shown in Fig. 9a. After the first self-check, all devices were installed symmetrically near the central axis of the mounting plate, which is made of wood and aluminum. All cables were tightened to eliminate any micro-vibration which would cause non-negligible interference to the measured acceleration (Fig. 9b). After completing the second self-check, the inner capsule was sealed and lifted up for barycentric correction (Fig. 9c). Additionally, by correcting the barycenter, the inner capsule was prevented from rotating during the free-fall.

Subsequently, when the barycentric correction was completed, the inner capsule was lifted into the outer capsule (Fig. 9d). Then, the outer capsule was pumped below 30 Pa and sealed (Fig. 9e). Before falling from the top of the tower, the vacuum pressure generally increased to around 80 Pa due to the slow air leakage effect.

Data were recorded from the beginning to the end of the experiment until the power of the inner capsule was cut off. In order to ensure that the power during the test was substantial, both the MCU and the data acquisition card were powered independently by lead batteries.

### Control strategy and parameter settings

In order to adapt to the microgravity level in the NMLC drop tower (10^−2^ mg_0_), the maximum feedback voltages of the SIS were amplified and the measurement range along the horizontal axes was expanded from ±5 μg_0_ to ±1.6 mg_0_ (large-range mode) and ±0.46 mg_0_ (small-range mode). At the same time, the ability to capture the test mass in a microgravity environment was enhanced, ensuring an adequate capturing and measurement ability for the drop tower experiment (Table 4). The maximum operational acceleration reflects the maximum residual acceleration at which the SIS can operate.

At the beginning of the free-fall, the test mass in the SIS rested on the bottom of the electrode housing. The primary task was to control the test mass to the center of the electrode housing. Therefore, prior to measurement, the MCU was in capture mode with the lowest position detection voltage (1 V), the highest bias voltage (55 V), and a maximum feedback voltage of up to 35 V. In this parameter configuration, the maximum operational acceleration was relatively large (Table 4).

Once the displacement voltages of all axes were within ±0.5 V and the duration exceeded 0.5 s, the large-range mode switching would begin and *V*_d_ would switch from 1 to 4 V to increase the stiffness and optimize the stability of the system.

In the small-range mode switching experiment, the mode switch would begin when the displacement voltages of all axes were within ±0.1 V for more than 0.5 s. After the mode switch, the bias voltage was reduced from 55 to 30 V, the gain of the feedback loop was reduced by half, and the position detection voltage (*V*_d_) was switched to 4 V.

By reducing the bias voltage and the gain of the feedback loop, the circuit noise was suppressed, improving the resolution of the measurement. This small-range mode allows for more accurate measurements when the residual acceleration of the satellite is further reduced.

### Mode switch simulation prior to free-fall

A single drop tower experiment may last 4~5 h and may fail for unpredictable reasons. To ensure the success of the experiment, the Simulink software (https://ww2.mathworks.cn) was used to simulate the control stability of the system during the fall and determine the optimal control parameters before and after the mode switch.

**Fig. 10:**
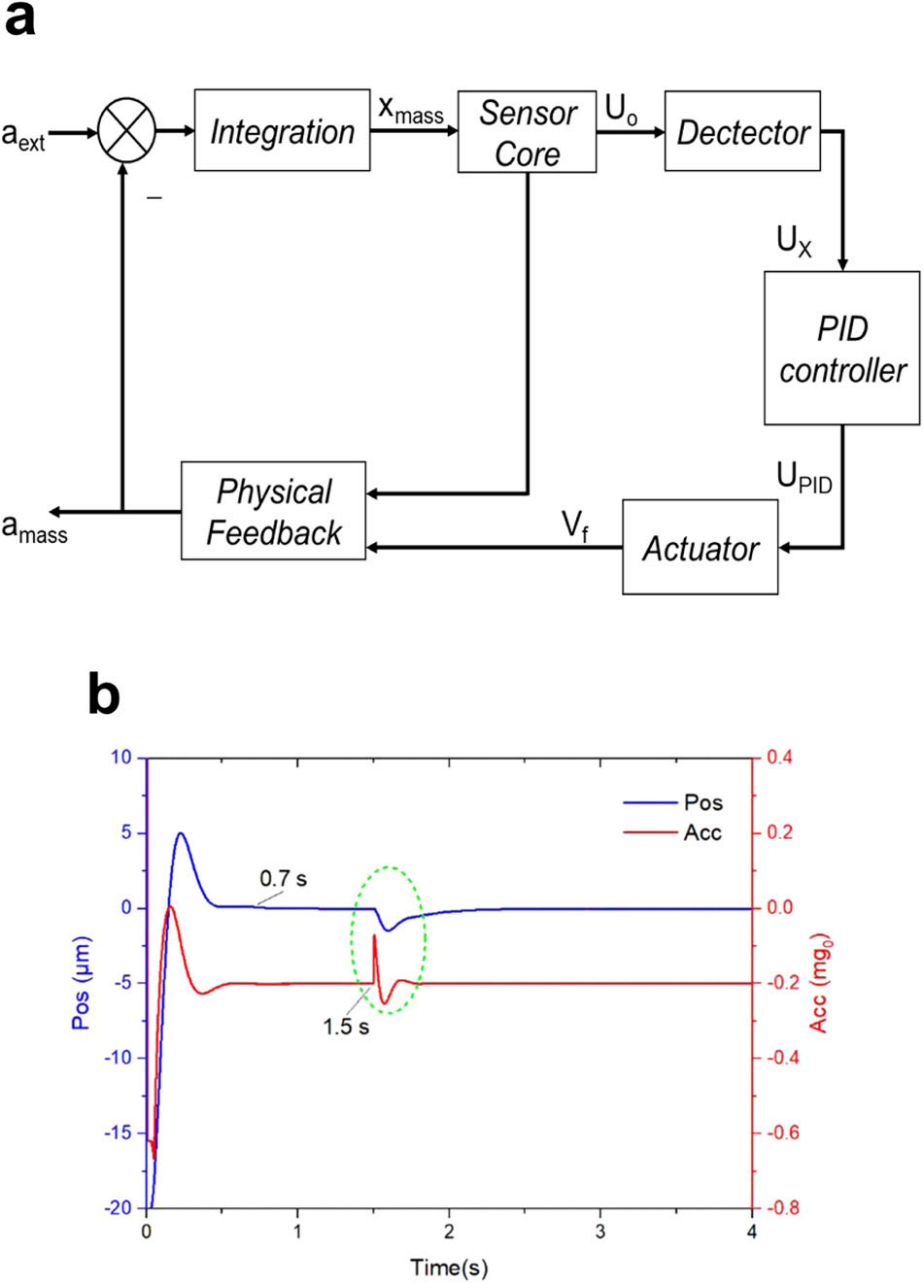
Simulation for the drop tower test. **a** Uniaxial simulation model of the SIS. **b** Displacement and acceleration along the Y-axis in small-range mode switching experiment.

The uniaxial simulation model for the drop tower test is illustrated in Fig. 10a, where *a*_ext_ is the external residual acceleration, *x*_mass_ is the displacement of the test mass, *U*_o_ is the output voltage of the charge amplifier, *V*_f_ is the feedback voltage, and *a*_mass_ is the acceleration produced by the electrostatic force.

In the simulation model, the noise, gain, and delay in the real circuit were taken into consideration. According to the microgravity level of the drop tower^21^, *a*_ext_ was set to a constant value of –2 × 10^−3^ m/s^2^ along the three axes of the model.

The PID controller plays a crucial role in a real circuit, since, when in-orbit, the test mass may get out of control if the key parameters of the PID controller are not set properly. The available time is limited since there are only about 3.5 s of free-fall during a drop test; thus, the effectiveness and correctness of the control loop are of particular importance.

The PID controller contains proportional, differential, and integral terms. Its transfer function^22,23^ is given by Eq. (3).

3 $$G\left( s \right) = K_{\mathrm{p}} + \frac{{K_{\mathrm{I}}}}{s} + K_{\mathrm{D}}s$$

where *K*_p_ is the proportionality coefficient, *K*_I_ is the integral term coefficient, *K*_D_ is the differential term coefficient, and *s* is the Laplace operator.

In the simulation, *K*_p_, *K*_I_, and *K*_D_ need to be fine-tuned so that a sufficient gain and phase margin can be obtained for a stable system.

In the uniaxial simulation model, an electrostatic force equation was used, as shown in Eq. (4)^24^.

4 $$F_{{\mathrm{el}}} = 2\varepsilon _0S\frac{{V_{\mathrm{p}}V_{\mathrm{f}}(d^2 + x^2) - \left( {V_{\mathrm{f}}^2 + V_{\mathrm{p}}^2 + V_{\mathrm{d}}^2} \right) \cdot d \cdot x}}{{(d^2 - x^2)^2}}$$

where *S* is the area of the electrode, *ε*_0_ is the permittivity of vacuum, *V*_p_ is the bias voltage, *V*_f_ is the feedback voltage, *V*_d_ is the detection voltage, *d* is the average distance between electrode and test mass when the test mass is at the center of the electrode housing, and *x* is the displacement of test mass relative to the center of the electrode housing.

According to Eq. (4), the electrostatic force applied on the test mass can be calculated from the real-time feedback voltage (*V*_f_) and displacement (*x*).

The simulation results of the displacement and acceleration along the Y-axis in the small-range mode switching test were taken as an example, as shown in Fig. 10b. It can be seen that at about 0.7 s after release, the test mass can be controlled at the center of the electrode housing. The mode switch took place at 1.5 s, causing a vibration of up to 0.55 μm, and an acceleration overshoot of about 0.21 mg_0_, as indicated by the circled area. After mode switching, the displacement curve converged to zero within 0.7 s.

The other axes were simulated and optimized in the same way to ensure that the test mass could be captured within 1 s and the displacement vibration would remain within ±1 μm after mode switching. The experimental results show that a proper simulation before a drop test is a prerequisite for the success of the test.

### Reporting summary

Further information on research design is available in the Nature Research Reporting Summary linked to this article.

## Supplementary information

Reporting summary.

## Data Availability

The datasets generated and analyzed during the current study are available from the corresponding author on reasonable request.
